# A nationwide study on sleep complaints and associated factors in older adults: ELSI-Brazil

**DOI:** 10.1590/0102-311XEN061923

**Published:** 2023-11-10

**Authors:** Jaquelini Betta Canever, Letícia Martins Cândido, Bruno de Souza Moreira, Ana Lúcia Danielewicz, Helena Iturvides Cimarosti, Maria Fernanda Lima-Costa, Núbia Carelli Pereira de Avelar

**Affiliations:** 1 Centro de Ciências, Tecnologias e Saúde do Campus Araranguá, Universidade Federal de Santa Catarina, Araranguá, Brasil.; 2 Núcleo de Estudos em Saúde Pública e Envelhecimento, Fundação Oswaldo Cruz/Universidade Federal de Minas Gerais, Belo Horizonte, Brasil.; 3 Universidade Federal de Santa Catarina, Florianópolis, Brasil.

**Keywords:** Daytime Sleepiness, Insomnia, Aged, Prevalence, Sleep Quality, Sonolência Diurna, Insônia, Idoso, Prevalência, Qualidade do Sono, Somnolencia Diurna, Insomnio, Anciano, Prevalencia, Calidad del Sueño

## Abstract

Sleep problems, such as difficulty falling asleep, staying asleep, early awakening with failure to continue sleep, and altered sleep-wake cycle, are common in the general population. This cross-sectional study with 6,929 older adults (≥ 60 years) aimed to estimate the prevalence of different types of sleep problems, their associated factors, and the population-attributable fraction of associated factors among older adults. The outcome variables consisted of self-reported sleep problems: insomnia (initial, intermediate, late, and any type of insomnia), poor sleep quality, and daytime sleepiness. The independent variables were sociodemographic and behavioral characteristics and health conditions. The prevalence proportions were initial insomnia (49.1%), intermediate insomnia (49.2%), late insomnia (45.9%), any type of insomnia (58.6%), poor sleep quality (15.6%), and daytime sleepiness (38.4%). Female sex, presence of two or more chronic diseases, not eating the recommended amount of fruits and vegetables, and regular and bad/very bad self-rated health were positively associated with the sleep problems investigated. Consuming alcohol once a month or more was inversely associated with initial insomnia. Population attributable fraction estimates ranged from 3% to 19% considering two or more chronic diseases, not eating the recommended amount of fruits and vegetables, and regular and bad/very bad self-rated health. High prevalence of self-reported sleep problems was evinced in older adults. These results can be useful to guide public health services in the creation of informational, evaluative, and screening strategies for sleep problems in older Brazilian adults.

## Resumo

Problemas de sono, como dificuldade para adormecer, permanecer dormindo, despertar precoce com falha na continuidade do sono e alteração do ciclo vigília-sono, são comuns na população em geral. Este estudo transversal com 6.929 idosos (≥ 60 anos) buscou estimar a prevalência de diferentes tipos de problemas de sono, seus fatores associados e a fração atribuível populacional de fatores associados a problemas de sono nessa população. As variáveis de desfecho foram problemas de sono autorreferidos: insônia (inicial, intermediária, tardia e qualquer tipo de insônia), má qualidade do sono e sonolência diurna. As variáveis independentes incluíram características sociodemográficas, comportamentais e condições de saúde. As proporções de prevalência foram: insônia inicial (49,1%), insônia intermediária (49,2%), insônia tardia (45,9%), qualquer tipo de insônia (58,6%), má qualidade do sono (15,6%) e sonolência diurna (38,4%). Sexo feminino, presença de duas ou mais doenças crônicas, não consumir a quantidade recomendada de frutas e hortaliças e autoavaliação da saúde como regular e ruim/muito ruim mostraram associação positiva aos problemas de sono investigados. Consumo de álcool uma vez por mês ou mais associou-se inversamente à insônia inicial. As estimativas da fração atribuível populacional variaram de 3% a 19% considerando duas ou mais doenças crônicas, consumo insuficiente de frutas e vegetais e saúde autorrelatada regular/ruim/muito ruim. Evidenciou-se alta prevalência de problemas de sono autorreferidos em idosos. Esses resultados podem orientar os serviços públicos de saúde na criação de estratégias informativas, avaliativas e de rastreamento de problemas de sono em idosos brasileiros.

## Resumen

Problemas del sueño, como la dificultad para conciliar el sueño, permanecer dormido, despertarse temprano sin poder seguir durmiendo y cambios en el ciclo de sueño y vigilia, son comunes en la población en general. Este estudio transversal con 6.929 personas mayores (≥ 60 años) buscó estimar la prevalencia de diferentes tipos de problemas de sueño, sus factores asociados y la fracción atribuible a la población de factores asociados con problemas de sueño en esta población. Las variables de desenlace fueron problemas de sueño autoinformados: insomnio (inicial, intermedio, tardío y cualquier tipo de insomnio), mala calidad del sueño y somnolencia diurna. Las variables independientes incluyeron características sociodemográficos y conductuales y condiciones de salud. Estas fueron las proporciones de prevalencia: insomnio inicial (49,1%), insomnio intermedio (49,2%), insomnio tardío (45,9%), cualquier tipo de insomnio (58,6%), mala calidad del sueño (15,6%) y somnolencia diurna (38,4%). El sexo femenino, la presencia de dos o más enfermedades crónicas, no consumir la cantidad recomendada de frutas y hortalizas y la autoevaluación de la salud como regular y mala/muy mala mostraron una asociación positiva con los problemas de sueño investigados. El consumo de alcohol una vez al mes o más se asoció inversamente con el insomnio inicial. Las estimaciones de la fracción atribuible de la población oscilaron entre el 3% y el 19% considerando dos o más enfermedades crónicas, un consumo insuficiente de frutas y verduras y una salud autoinformada regular/mala/muy mala. Se evidenció una alta prevalencia de problemas de sueño autoinformados en las personas mayores. Estos resultados pueden orientar los servicios públicos de salud en la creación de estrategias informativas, evaluativas y de seguimiento de los problemas de sueño en las personas mayores brasileñas.

## Introduction

Sleep problems, such as difficulty falling asleep, staying asleep, early awakening with failure to continue sleep, are common in the general population ^1^. The prevalence of sleep problems has increased in recent years, affecting 35% to 70% of community-dwelling older adults worldwide ^1^ ^,^ ^2^. In Brazil, approximately 41.2% of older adults report sleep problems ^3^, while developed countries have shown even higher proportions, with a prevalence of 50% in Poland and 67% in Austria ^4^.

Sleep problems represent an expressive economic and social burden for the individual and the society ^5^. Estimates indicate that the annual expenditure on the treatment of these problems in developed countries, such as the United States, exceeds USD 94 billion ^6^. Moreover, sleep problems are associated with several negative health outcomes, such as heart and lung diseases ^7^, neurological and cognitive complaints ^8^, immune system dysfunctions ^9^, frailty ^10^, and mortality ^11^. Thus, the study of sleep problems in older adults is necessary since the information can increase the longevity and quality of life of individuals.

Some associated factors for sleep problems in older adults have already been identified, such as female sex ^12^, lack of partners ^13^, poor education ^14^, low socioeconomic status ^14^, use of medication (e.g., antidepressants) ^15^, chronic diseases ^16^, depression ^17^, and sedentary behavior ^3^.

Studies from China ^18^, Iran ^19^, and the United States ^20^ ^,^ ^21^ have focused on different typologies of sleep problems and associated factors in their respective populations. In Brazil, only one study has examined the factors associated with sleep problems among adults ^22^. However, the authors assessed sleep problems by a single screening question and examined only health variables as possible associated factors ^22^. Recently, sleep problems have been stratified into different typologies ^23^. Since the previous study conducted in Brazil did not address different sleep problems, this study aims to describe the prevalence proportions of each sleep problem typology, as well as their respective relationship with sociodemographic, behavioral, and health factors in older adults. Additionally, we calculated the population-attributable fraction (PAF) of potentially modifiable associated factors of sleep problems.

## Methods

### Study design

This was a cross-sectional study conducted with data from the second wave of the *Brazilian Longitudinal Study of Aging* (ELSI-Brazil). ELSI-Brazil is a nationally based study conducted with community-dwelling adults aged 50 years and over. All residents aged 50 years and over in the selected households were eligible for interviews and physical assessments. The final sample included individuals living in 70 municipalities from the five Brazilian macroregions. The second wave of the survey was conducted from August 2019 to March 2021 and included 9,949 participants. Data were obtained in face-to-face interviews conducted at participants’ homes. More details about the sampling, methodology, and national representativeness of ELSI-Brazil can be found in previous publications ^24^ ^,^ ^25^. For this study, only data from participants aged 60 years and over were considered in the analyses. This age is the cutoff point used for classifying an individual as an older adult in developing countries, such as Brazil. ELSI-Brazil was approved by the Research Ethics Committee of the René Rachou Institute, Oswaldo Cruz Foundation (CAAE: 34649814.3.0000.5091).

### Outcomes

The outcomes of this study were the different typologies of sleep problems, assessed by self-report: initial insomnia ^26^, intermediate insomnia ^27^, late insomnia ^27^, any type of insomnia ^27^, poor sleep quality ^28^, and daytime sleepiness ^29^.

The initial, intermediate, and late insomnia were assessed by the following questions, respectively: “How often do you have problems falling asleep (lying down and sleeping)?”, “How often do you have sleeping problems because you wake up during the night?”, and “How often do you have sleeping problems because you wake up early and cannot go back to sleep?”, with the response options: (1) most of the time; (2) sometimes; and (3) never/rarely. The variables for assessing insomnia were recategorized into presence of insomnia (response options 1 and 2) and absence of insomnia (response option 3) ^23^. Subsequently, “any type of insomnia” variable was created, which consisted of the presence of at least one type of insomnia (initial, intermediate, or late insomnia).

The quality of sleep was evaluated by the question: “How do you evaluate the quality of your sleep?”, with the response options: (1) very good; (2) good; (3) regular; (4) poor; and (5) very poor. This variable was recategorized into poor quality of sleep (response options 4 and 5) and good quality of sleep (response options 1, 2, and 3) ^28^.

The variable daytime sleepiness was assessed by the question: “How often do you wake up rested in the morning?”, with the response options: (1) most of the time; (2) sometimes; and (3) never/rarely. This variable was recategorized into absence of daytime sleepiness (response option 1) and presence of daytime sleepiness (response options 2 and 3).

### Independent variables

Sociodemographic characteristics included sex (female or male), age group in years (60-69, 70-79, or ≥ 80), years of schooling (illiterate, 1-4, 5-8 or ≥ 9), monthly income in minimum wages (no income, < 2, 2-5, or > 5), and marital status (not married - i.e., single, divorced/separated, or widowed - or married/stable union).

Behavioral characteristics included alcohol intake (does not consume alcoholic beverages, consumes less than once a month, or consumes once a month or more), smoking (never smoked, former smoker, or current smoker), adequate consumption of fruits (including natural juice) and vegetables (no or yes), total sedentary behavior (< 3 hours per day, 3-6 hours per day, or > 6 hours per day), and level of leisure-time physical activity (insufficiently active or sufficiently active). Adequate consumption of fruits and vegetables consisted of consuming at least 25 portions of these foods per week, considering the sum of these portions, which is approximately equivalent to the daily consumption of five portions of these foods. Total sedentary behavior was determined based on the weighted average of sitting time on a weekday and on a weekend day [(time in the week × 5) + (time at the weekend × 2)] / 7). For the level of leisure-time physical activity, participants who performed > 150 minutes of walking and moderate-intensity physical activity per week or > 75 minutes of vigorous-intensity physical activity per week were considered sufficiently active, whereas those who performed these activities with a shorter weekly duration were considered insufficiently active.

Assessed health conditions included nutritional status defined by body mass index - BMI in kg/m^2^ (underweight < 22.0, eutrophic 22.0-27.0, or overweight > 27.0) ^30^, number of chronic diseases diagnosed by a physician based on self-report (0, 1, or ≥ 2), and self-rated health (excellent/very good/good, regular, or bad/very bad). The following chronic diseases were investigated: hypertension, diabetes mellitus, hypercholesterolemia, myocardial infarction, angina pectoris, heart failure, stroke, asthma, chronic obstructive pulmonary disease, arthritis or rheumatism, osteoporosis, chronic back problems, depression, cancer, chronic renal failure, Parkinson’s disease, and Alzheimer’s disease.

### Statistical analysis

The software Stata, version 14.0 (https://www.stata.com), was used to analyze the data. The effect of sample design and individual weights were incorporated into all analyses using the *svy* command. Prevalence proportions and 95% confidence interval (95%CI) for each sleep problem for the study population and stratified by sex were plotted in graphs. The association between each typology of sleep problem and the independent variables was investigated using logistic regression, estimating odds ratios (OR) and their respective 95%CI. Independent variables presenting unadjusted association with p-value < 0.20 (Wald test) were included in the adjusted models.

Then, adjusted logistic regression analysis for each sleep problem was performed with a hierarchical entry of the independent variables in three stages according to the theoretical model ^31^ ^,^ ^32^. In the first stage, regression analysis was performed with the sociodemographic characteristics selected from the unadjusted analysis with a p-value < 0.20. In the second stage, the behavioral characteristics selected from the unadjusted analysis with a p-value < 0.20 were introduced along with the significant sociodemographic characteristics with a p-value < 0.05 from the first stage. In the third stage, the health conditions selected from the unadjusted analysis with a p-value < 0.20 were added with the significant sociodemographic and behavioral characteristics with a p-value < 0.05 from the second stage.

Furthermore, the PAF for potentially modifiable associated factors (behavioral characteristics and health conditions) for the occurrence of any type of insomnia, poor sleep quality, and daytime sleepiness was estimated. For this purpose, the *regpar* command of the software Stata was used.

## Results

**Figure 1:**
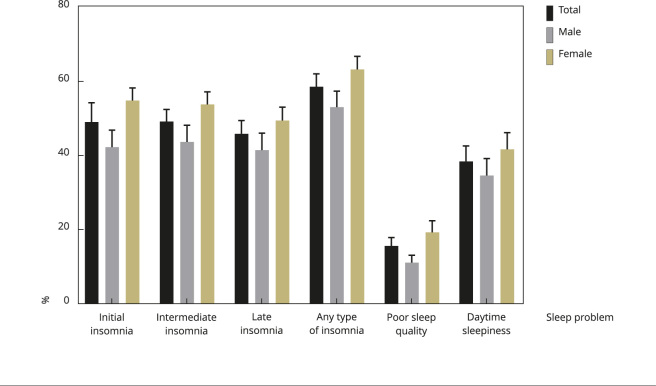
Prevalence (%) and standard deviation for each sleep problem among the study population stratified by sex. *Brazilian Longitudinal Study of Aging* (ELSI-Brazil), 2019-2021.

Data from 6,929 older adults (71.0 ± 8.2 years) were analyzed in this study. The prevalence proportions of initial, intermediate, late insomnia, and any type of insomnia were 49.1% (95%CI: 45.6; 52.7), 49.2% (95%CI: 45.9; 52.5), 45.9% (95%CI: 42.3; 49.5), and 58.6% (95%CI: 55.1; 62.1), respectively (Figure 1). Regarding the other typologies of sleep problems, the prevalence was 15.6% (95%CI: 13.5; 17.9) for poor sleep quality and 38.4% (95%CI: 34.4; 42.6) for daytime sleepiness (Figure 1). Tables 1 and 2 present the prevalence proportions and 95%CI for sleep problems according to sociodemographic and behavioral characteristics and health conditions, as well as the results of the unadjusted association analyses.

**Table 1:** Sample description and unadjusted association of initial, intermediate, and late insomnia with sociodemographic and behavioral characteristics and health conditions in older adults (≥ 60 years). *Brazilian Longitudinal Study of Aging* (ELSI-Brazil), 2019-2021.

Characteristics	Initial insomnia		Intermediate insomnia		Late insomnia	
	% (95%CI)	Unadjusted OR (95%CI)	% (95%CI)	Unadjusted OR (95%CI)	% (95%CI)	Unadjusted OR (95%CI)
Sociodemographic						
Sex [n = 6,929]						
Male	42.3 (37.8; 46.9)	Reference	43.7 (39.4; 48.2)	Reference	41.5 (37.1; 46.1)	Reference
Female	54.9 (51.5; 58.3)	1.65 (1.41; 1.94) *	53.8 (50.4; 57.2)	1.50 (1.26; 1.78) *	49.5 (46.0; 53.1)	1.38 (1.18; 1.61) *
Age group (years) [n = 6,929]						
60-69	47.9 (44.0; 51.9)	Reference	47.6 (43.9; 51.4)	Reference	43.6 (39.4; 47.8)	Reference
70-79	49.6 (45.1; 54.2)	1.07 (0.88; 1.29) *	50.1 (45.8; 54.3)	1.10 (0.91; 1.32) *	47.0 (42.5; 51.5)	1.14 (0.94; 1.40) *
≥ 80	52.7 (47.1; 58.3)	1.21 (1.00; 1.45) *	53.4 (47.8; 58.9)	1.26 (1.05; 1.50) *	52.6 (47.3; 57.8)	1.43 (1.17; 1.75) *
Years of schooling [n = 6,807]						
≥ 9	43.2 (38.2; 48.3)	Reference	43.8 (38.5; 49.2)	Reference	38.7 (33.8; 43.8)	Reference
5-8	46.4 (41.2; 51.8)	1.14 (0.94; 2.18) *	49.2 (44.1; 54.3)	1.24 (1.03; 1.49) *	44.5 (38.8; 50.3)	1.26 (1.04; 1.54) *
1-4	50.7 (46.0; 55.3)	1.35 (1.10; 1.65) *	49.6 (45.7; 53.6)	1.26 (1.03; 1.54) *	47.5 (43.5; 51.6)	1.43 (1.20; 1.71) *
Illiterate	55.2 (49.1; 61.2)	1.62 (1.20; 2.18) *	54.2 (48.5; 59.8)	1.51 (1.13; 2.04) *	51.3 (45.0; 57.6)	1.67 (1.25; 2.22) *
Monthly income (minimum wages) ** [n = 6,929]						
> 5	37.6 (26.0; 50.9)	Reference	34.3 (20.3; 51.6)	Reference	31.3 (17.0; 50.4)	Reference
2-5	40.3 (32.9; 48.1)	1.11 (0.67; 1.84) *	43.3 (36.4; 50.6)	1.46 (0.80; 2.67) *	39.4 (32.1; 47.2)	1.42 (0.72; 2.80) *
< 2	51.0 (47.4; 54.5)	1.72 (1.06; 2.79) *	50.9 (47.7; 54.0)	1.98 (1.09; 3.62) *	47.8 (43.9; 51.6)	2.00 (1.01; 3.95) *
No income	50.6 (43.3; 57.8)	1.69 (0.99; 2.89) *	49.4 (42.2; 56.7)	1.87 (1.12; 3.14) *	45.8 (39.1; 52.6)	1.85 (1.02; 3.34) *
Marital status [n = 6,929]						
Married/Stable union	47.2 (43.3; 51.2)	Reference	47.4 (43.8; 51.1)	Reference	43.6 (39.6; 47.7)	Reference
Not married	51.6 (47.6; 55.6)	1.19 (1.02; 1.39) *	51.5 (47.6; 55.4)	1.17 (1.01; 1.35) *	48.9 (44.9; 53.0)	1.23 (1.06; 1.44) *
Behavioral						
Alcohol intake (times per month) [n = 6,870]						
No consumption	51.7 (48.2; 55.3)	Reference	51.0 (47.7; 54.4)	Reference	48.2 (44.6; 51.9)	Reference
< 1	44.3 (35.6; 53.4)	0.74 (0.54; 1.00) *	44.6 (35.6; 53.9)	0.77 (0.57; 1.04) *	39.6 (30.6; 49.3)	0.70 (0.51; 0.96) *
≥ 1	35.8 (29.4; 42.7)	0.51 (0.39; 0.67) *	40.3 (33.7; 47.3)	0.64 (0.49; 0.85) *	34.9 (27.7; 42.9)	0.57 (0.41; 0.79) *
Smoking [n = 6,915]						
Never smoked	50.4 (46.4; 54.4)	Reference	50.4 (46.5; 54.3)	Reference	47.6 (43.3; 51.9)	Reference
Former smoker	45.2 (41.3; 49.2)	0.81 (0.69; 0.95) *	45.7 (41.6; 50.0)	0.83 (0.68; 1.01) *	41.3 (37.2; 45.5)	0.77 (0.63; 0.95) *
Current smoker	50.8 (43.9; 57.6)	1.01 (0.78; 1.31) *	50.2 (43.5; 56.9)	0.99 (0.79; 1.24) *	46.4 (39.9; 52.9)	0.95 (0.76; 1.14) *
Adequate consumption of fruits and vegetables *** [n = 6,790]						
Yes	44.5 (37.8; 51.5)	Reference	46.6 (40.3; 53.0)	Reference	43.7 (36.7; 51.0)	Reference
No	50.4 (46.6; 54.1)	1.26 (0.96; 1.65) *	49.8 (46.4; 53.2)	1.13 (0.88; 1.46)	46.4 (42.8; 50.1)	1.11 (0.84; 1.47)
Total sedentary behavior (hours per day) ^#^ [n = 6,322]						
< 3	46.6 (42.1; 51.2)	Reference	46.8 (42.6; 51.1)	Reference	43.6 (39.2; 48.2)	Reference
3-6	49.6 (44.9; 54.2)	1.12 (0.89; 1.40)	50.4 (46.3; 54.6)	1.15 (0.95; 1.39)	46.0 (41.5; 50.6)	1.10 (0.88; 1.36) *
> 6	53.0 (46.5; 59.4)	1.28 (0.97; 1.70)	52.6 (46.3; 58.8)	1.25 (0.95; 1.66)	50.6 (43.9; 57.3)	1.32 (0.99; 1.76) *
Level of leisure-time physical activity ^##^ [n = 6,929]						
Sufficiently active	46.3 (41.5; 51.2)	Reference	47.6 (42.6; 52.7)	Reference	44.6 (38.9; 50.5)	Reference
Insufficiently active	49.8 (46.1; 53.5)	1.14 (0.96; 1.36) *	49.6 (46.0; 53.1)	1.08 (0.88; 1.32)	46.2 (42.5; 49.9)	1.06 (0.87; 1.30)
Health conditions						
Nutritional status ^###^ [n = 5,843]						
Eutrophic	48.3 (44.7; 51.8)	Reference	48.0 (44.3; 51.8)	Reference	44.8 (40.9; 48.8)	Reference
Underweight	50.6 (44.0; 57.3)	1.09 (0.85; 1.41)	48.7 (42.8; 54.8)	1.02 (0.79; 1.32)	48.6 (42.4; 54.9)	1.16 (0.89; 1.50)
Overweight	48.7 (45.1; 52.3)	1.01 (0.87; 1.18)	49.5 (46.1; 52.9)	1.06 (0.89; 1.25)	45.1 (41.8; 48.5)	1.01 (0.89; 1.14)
Number of chronic diseases ^§^ [n = 6,656]						
0	40.4 (36.1; 44.9)	Reference	35.1 (30.7; 39.7)	Reference	34.9 (30.3; 39.9)	Reference
1	41.7 (37.1; 46.5)	1.05 (0.87; 1.27) *	41.7 (37.7; 45.9)	1.32 (1.09; 1.60) *	40.1 (35.4; 44.9)	1.24 (1.03; 1.49) *
≥ 2	54.7 (50.7; 58.7)	1.78 (1.49; 2.12) *	56.1 (52.2; 59.9)	2.36 (1.92; 2.89) *	51.4 (47.2; 55.5)	1.96 (1.59; 2.42) *
Self-rated health [n = 6,887]						
Excellent/Very good/Good	38.2 (33.9; 42.7)	Reference	36.4 (32.1; 40.9)	Reference	33.9 (29.8; 38.3)	Reference
Regular	52.9 (48.7; 57.1)	1.81 (1.47; 2.24) *	54.8 (50.6; 58.9)	2.11 (1.69; 2.63) *	49.8 (45.2; 54.5)	1.93 (1.54; 2.41) *
Bad/Very bad	68.4 (63.0; 73.3)	3.49 (2.75; 4.44) *	69.3 (64.8; 73.4)	3.94 (3.05; 5.09) *	67.5 (62.3; 72.2)	4.04 (3.14; 5.18) *
N (unweighted)	6,849		6,846		6,839	

95%CI: 95% confidence interval; OR: odds ratio (estimated by logistic regression).

Note: all estimates considered the weights of the individuals and the complex sample design.

* p-value < 0.20;

** Minimum wage during 2019-2021 was BRL 1,212;

*** Adequate consumption of fruits (including natural juice) and vegetables consisted of consuming at least 25 portions of these foods per week, considering the sum of these portions, which is approximately equivalent to the daily consumption of five portions;

^#^ Total sedentary behavior was determined based on the weighted average of the time spent sitting on a weekday and on a weekend day [(time during the week × 5) + (time during the weekend × 2)] / 7);

^##^ Level of leisure-time physical activity: insufficiently active (< 150 minutes of walking and moderate-intensity physical activity per week or < 75 minutes of vigorous-intensity physical activity per week) and sufficiently active (> 150 minutes of walking and moderate-intensity physical activity per week or > 75 minutes of vigorous-intensity physical activity per week);

^###^ Nutritional status was defined by body mass index [underweight (< 22.0kg/m^2^), eutrophic (22.0 to 27.0kg/m^2^), and overweight (> 27.0kg/m^2^)];

^§^ Chronic diseases included hypertension, diabetes mellitus, hypercholesterolemia, myocardial infarction, angina pectoris, heart failure, stroke, asthma, chronic obstructive pulmonary disease, arthritis or rheumatism, osteoporosis, chronic back problems, depression, cancer, chronic renal failure, Parkinson’s disease, and Alzheimer’s disease.

**Table 2:** Sample description and unadjusted association of any type of insomnia, poor sleep quality, and daytime sleepiness with sociodemographic and behavioral characteristics and health conditions in older adults (≥ 60 years). *Brazilian Longitudinal Study of Aging* (ELSI-Brazil), 2019-2021.

Characteristics	Any type of insomnia		Poor sleep quality		Daytime sleepiness	
	% (95%CI)	Unadjusted OR (95%CI)	% (95%CI)	Unadjusted OR (95%CI)	% (95%CI)	Unadjusted OR (95%CI)
Sociodemographic						
Sex [n = 6,929]						
Male	53.1 (48.7; 57.4)	Reference	11.1 (9.3; 13.1)	Reference	34.6 (30.3; 39.2)	Reference
Female	63.3 (59.7; 66.8)	1.52 (1.29; 1.79) *	19.3 (16.6; 22.4)	1.91 (1.61; 2.27) *	41.7 (37.3; 46.2)	1.34 (1.15; 1.57) *
Age group (years) [n = 6,929]						
60-69	56.9 (52.8; 61.0)	Reference	15.3 (13.2; 17.7)	Reference	39.0 (34.4; 43.7)	Reference
70-79	60.1 (55.7; 64.2)	1.13 (0.93; 1.38) *	15.7 (12.8; 19.0)	1.02 (0.83; 1.27)	37.4 (33.4; 41.5)	0.93 (0.80; 1.08)
≥ 80	62.3 (56.7; 67.5)	1.24 (1.02; 1.51) *	16.5 (12.4; 21.6)	1.09 (0.81; 1.46)	38.8 (32.6; 45.3)	0.99 (0.82; 1.08)
Years of schooling [n = 6,807]						
≥ 9	53.3 (48.0; 58.5)	Reference	13.4 (9.9; 17.8)	Reference	33.3 (28.3; 38.7)	Reference
5-8	58.9 (53.4; 64.3)	1.25 (0.01; 1.55) *	12.6 (9.1; 17.1)	0.93 (0.64; 1.34) *	37.9 (30.8; 45.5)	1.22 (0.96; 1.55) *
1-4	59.6 (55.3; 63.7)	1.29 (1.07; 1.55) *	16.3 (13.9; 19.0)	1.26 (0.94; 1.69) *	40.1 (35.5; 44.8)	1.34 (1.07; 1.67) *
Illiterate	62.0 (56.2; 67.4)	1.42 (1.04; 1.94) *	20.3 (16.9; 24.1)	1.64 (1.15; 2.35) *	41.0 (33.0; 49.5)	1.39 (0.96; 2.02) *
Monthly income (minimum wages) ** [n = 6,929]						
> 5	48.1 (31.2; 65.5)	Reference	5.5 (1.8; 15.2)	Reference	29.5 (18.5; 43.5)	Reference
2-5	53.8 (46.4; 61.0)	1.25 (0.68; 2.27) *	10.4 (8.0; 13.4)	1.97 (0.74; 5.21) *	31.9 (24.7; 40.0)	1.11 (0.62; 1.98) *
< 2	60.1 (56.6; 63.5)	1.62 (0.88; 2.95) *	17.6 (15.1; 20.3)	3.61 (1.50; 8.67) *	39.6 (35.5; 43.7)	1.56 (0.91; 2.66) *
No income	58.2 (51.4; 64.8)	1.50 (0.83; 2.70) *	12.8 (9.5; 17.1)	2.50 (1.02; 6.09) *	40.7 (33.4; 48.4)	1.64 (0.95; 2.83) *
Marital status [n = 6,929]						
Married/Stable union	57.3 (53.5; 61.0)	Reference	14.3 (12.5; 16.4)	Reference	37.1 (32.9; 41.4)	Reference
Not married	60.4 (56.4; 64.3)	1.13 (0.99; 1.30) *	17.2 (14.3; 20.6)	1.24 (1.05; 1.47) *	40.3 (34.8; 46.0)	1.14 (0.91; 1.43)
Behavioral						
Alcohol intake (times per month) [n = 6,870]						
No consumption	60.6 (57.0; 64.1)	Reference	16.7 (14.2; 19.5)	Reference	40.1 (35.7; 44.6)	Reference
< 1	53.4 (43.1; 63.4)	0.74 (0.53; 1.04) *	10.6 (7.0; 15.8)	0.59 (0.39; 0.88) *	34.8 (27.2; 43.3)	0.80 (0.59; 1.08) *
≥ 1	49.2 (42.6; 55.8)	0.62 (0.48; 0.81) *	11.8 (8.5; 16.1)	0.66 (0.45; 0.97) *	30.3 (24.2; 37.3)	0.65 (0.48; 0.87) *
Smoking [n = 6,915]						
Never smoked	59.7 (55.8; 63.5)	Reference	15.1 (12.9; 17.5)	Reference	40.1 (35.3; 45.2)	Reference
Former smoker	55.5 (51.4; 59.6)	0.84 (0.72; 0.98) *	17.0 (14.2; 20.2)	1.15 (0.96; 1.37)	34.8 (30.5; 39.4)	0.79 (0.62; 1.01) *
Current smoker	59.8 (52.6; 66.6)	1.00 (0.78; 1.28) *	15.2 (10.6; 21.4)	1.01 (0.69; 1.47)	36.9 (30.0; 44.3)	0.87 (0.69; 1.09) *
Adequate consumption of fruits and vegetables *** [n = 6,790]						
Yes	55.4 (48.8; 61.8)	Reference	11.9 (9.5; 14.9)	Reference	35.0 (26.2; 45.0)	Reference
No	59.4 (55.7; 63.0)	1.17 (0.90; 1.52)	16.5 (14.3; 19.1)	1.45 (1.14; 1.86) *	39.2 (35.3; 43.2)	1.19 (0.81; 1.74)
Total sedentary behavior (hours per day) ^#^ [n = 6,322]						
< 3	57.0 (52.2; 61.5)	Reference	14.2 (12.1; 16.6)	Reference	35.4 (31.4; 39.6)	Reference
3-6	58.5 (54.0; 62.9)	1.06 (0.86; 1.31)	16.3 (13.2; 19.9)	1.17 (0.93; 1.47) *	39.2 (34.0; 44.5)	1.17 (0.92; 1.49) *
> 6	62.5 (56.5; 68.2)	1.25 (0.94; 1.67)	17.3 (13.6; 21.8)	1.25 (0.97; 1.62) *	42.8 (33.6; 52.6)	1.36 (0.95; 1.95) *
Level of leisure-time physical activity ^##^ [n = 6,929]						
Sufficiently active	58.6 (53.5; 63.5)	Reference	14.1 (11.6; 17.1)	Reference	36.5 (30.3; 43.2)	Reference
Insufficiently active	58.7 (54.8; 62.4)	1.00 (0.80; 1.24)	15.9 (13.5; 18.6)	1.15 (0.89; 1.48)	38.9 (34.8; 43.1)	1.10 (0.88; 1.38)
Health conditions						
Nutritional status ^###^ [n = 5,843]						
Eutrophic	58.4 (54.5; 62.2)	Reference	15.5 (13.0; 18.4)	Reference	37.3 (33.0; 41.8)	Reference
Underweight	57.7 (51.4; 63.7)	0.97 (0.74; 1.27)	17.4 (13.8; 21.6)	1.14 (0.84; 1.56)	39.5 (34.0; 45.3)	1.10 (0.84; 1.44)
Overweight	58.9 (55.1; 62.7)	1.02 (0.87; 1.19)	15.8 (13.3; 18.7)	1.02 (0.85; 1.22)	37.8 (33.4; 42.5)	1.02 (0.86; 1.21)
Number of chronic diseases ^§^ [n = 6,656]						
0	46.9 (42.0; 51.7)	Reference	6.2 (4.4; 8.7)	Reference	31.9 (23.9; 41.2)	Reference
1	50.6 (46.1; 55.0)	1.16 (0.95; 1.40) *	9.7 (7.5; 12.4)	1.62 (1.02; 2.57) *	33.3 (28.9; 38.1)	1.06 (0.78; 1.42) *
≥ 2	65.2 (61.3; 69.0)	2.12 (1.73; 2.60) *	20.3 (17.8; 23.0)	3.82 (2.59; 5.64) *	42.0 (37.9; 46.3)	1.54 (1.08; 2.19) *
Self-rated health [n = 6,887]						
Excellent/Very good/Good	47.0 (42.5; 51.6)	Reference	7.2 (5.7; 9.2)	Reference	27.1 (23.0; 31.7)	Reference
Regular	63.7 (59.6; 67.7)	1.97 (1.61; 2.43) *	16.6 (14.0; 19.6)	2.54 (1.93; 3.34) *	43.7 (38.9; 48.6)	2.08 (1.61; 2.68) *
Bad/Very bad	76.9 (72.7; 80.5)	3.74 (2.95; 4.75) *	34.3 (29.8; 39.0)	6.65 (4.90; 9.03) *	55.6 (49.0; 62.0)	3.36 (2.61; 4.32) *
N (unweighted)	6,824		6,854		6,836	

95%CI: 95% confidence interval; OR: odds ratio (estimated by logistic regression).

Note: all estimates considered the weights of the individuals and the complex sample design.

* p-value < 0.20;

** Minimum wage during 2019-2021 was BRL 1,212;

*** Adequate consumption of fruits (including natural juice) and vegetables consisted of consuming at least 25 portions of these foods per week, considering the sum of these portions, which is approximately equivalent to the daily consumption of five portions;

^#^ Total sedentary behavior was determined based on the weighted average of the time spent sitting on a weekday and on a weekend day [(time during the week × 5) + (time during the weekend × 2)] / 7);

^##^ Level of leisure-time physical activity: insufficiently active (< 150 minutes of walking and moderate-intensity physical activity per week or < 75 minutes of vigorous-intensity physical activity per week) and sufficiently active (> 150 minutes of walking and moderate-intensity physical activity per week or > 75 minutes of vigorous-intensity physical activity per week);

^###^ Nutritional status was defined by body mass index [underweight (< 22.0kg/m^2^), eutrophic (22.0 to 27.0kg/m^2^), and overweight (> 27.0kg/m^2^)];

^§^ Chronic diseases included hypertension, diabetes mellitus, hypercholesterolemia, myocardial infarction, angina pectoris, heart failure, stroke, asthma, chronic obstructive pulmonary disease, arthritis or rheumatism, osteoporosis, chronic back problems, depression, cancer, chronic renal failure, Parkinson’s disease, and Alzheimer’s disease.

Among the sociodemographic characteristics, females were positively associated with initial insomnia (OR = 1.50; 95%CI: 1.27; 1.78), intermediate insomnia (OR = 1.36; 95%CI: 1.13; 1.63), late insomnia (OR = 1.25; 95%CI: 1.05; 1.48), any type of insomnia (OR = 1.37; 95%CI: 1.14; 1.65), poor sleep quality (OR = 1.72; 95%CI: 1.41; 2.11), and daytime sleepiness (OR = 1.31; 95%CI: 1.08; 1.58) (Table 3). For the behavioral characteristics, consuming alcohol once a month or more was inversely associated with initial insomnia (OR = 0.72; 95%CI: 0.53; 0.97), whereas not consuming the recommended amount of fruits and vegetables was positively associated with poor sleep quality (OR = 1.29; 95%CI: 1.03; 1.62) (Table 3).

**Table 3:** Final adjusted models of the factors associated with different typologies of sleep problems in older adults (≥ 60 years). *Brazilian Longitudinal Study of Aging* (ELSI-Brazil), 2019-2021.

Characteristics	Initial insomnia	Intermediate insomnia	Late insomnia	Any type of insomnia	Poor sleep quality	Daytime sleepiness
	Adjusted OR (95%CI)	Adjusted OR (95%CI)	Adjusted OR (95%CI)	Adjusted OR (95%CI)	Adjusted OR (95%CI)	Adjusted OR (95%CI)
Sociodemographic						
Sex [n = 6,929]						
Male	Reference	Reference	Reference	Reference	Reference	Reference
Female	1.50 (1.27; 1.78)	1.36 (1.13; 1.63)	1.25 (1.05; 1.48)	1.37 (1.14; 1.65)	1.72 (1.41; 2.11)	1.31 (1.08; 1.58)
Years of schooling [n = 6,807]						
≥ 9	Reference	-	Reference	-	-	Reference
5-8	1.04 (0.84; 1.29)	-	1.12 (0.91; 1.37)	-	-	1.11 (0.86; 1.42)
1-4	1.14 (0.92; 1.42)	-	1.15 (0.96; 1.39)	-	-	1.14 (0.92; 1.42)
Illiterate	1.18 (0.83; 1.68)	-	1.17 (0.84; 1.62)	-	-	1.05 (0.76; 1.44)
Monthly income (minimum wages) * [n = 6,929]						
> 5	-	-	-	-	Reference	-
2-5	-	-	-	-	1.48 (0.56; 3.95)	-
< 2	-	-	-	-	1.82 (0.77; 4.28)	-
No income	-	-	-	-	1.34 (0.53; 3.40)	-
Behavioral						
Alcohol intake (times per month) [n = 6,870]						
No consumption	Reference	Reference	Reference	Reference	-	-
< 1	0.97 (0.72; 1.31)	1.02 (0.78; 1.63)	0.92 (0.68; 1.24)	0.97 (0.70; 1.35)	-	-
≥ 1	0.72 (0.53; 0.97)	0.88 (0.65; 1.18)	0.78 (0.54; 1.10)	0.84 (0.63; 1.13)	-	-
Adequate consumption of fruits and vegetables ** [n = 6,790]						
Yes	-	-	-	-	Reference	-
No	-	-	-	-	1.29 (1.03; 1.62)	-
Health conditions						
Number of chronic diseases *** [n = 6,656]						
0	Reference	Reference	Reference	Reference	Reference	Reference
1	0.90 (0.74; 1.10)	1.16 (0.96; 1.41)	1.08 (0.91; 1.29)	1.01 (0.84; 1.23)	1.29 (0.80; 2.08)	0.93 (0.67; 1.28)
≥ 2	1.21 (1.01; 1.45)	1.65 (1.32; 2.05)	1.37 (1.12; 1.69)	1.49 (1.21; 1.84)	2.21 (1.44; 3.40)	1.06 (0.73; 1.55)
Self-rated health [n = 6,887]						
Excellent/Very good/Good	Reference	Reference	Reference	Reference	Reference	Reference
Regular	1.69 (1.36; 2.09)	1.88 (1.50; 2.37)	1.76 (1.42; 2.19)	1.80 (1.46; 2.22)	2.07 (1.57; 2.74)	2.00 (1.58; 2.52)
Bad/Very bad	3.00 (2.30; 3.91)	3.34 (2.55; 4.38)	3.45 (2.66; 4.47)	3.25 (2.49; 4.25)	4.97 (3.39; 7.29)	3.18 (2.43; 4.16)

95%CI: 95% confidence interval; OR: odds ratio (estimated by logistic regression).

Note: values in bold denote statistically significant association (p-value < 0.05); all estimates considered the weights of the individuals and the complex sample design.

* Minimum wage during 2019-2021 was BRL 1,212;

** Adequate consumption of fruits (including natural juice) and vegetables consisted of consuming at least 25 portions of these foods per week, considering the sum of these portions, which is approximately equivalent to the daily consumption of five portions;

*** Chronic diseases included hypertension, diabetes mellitus, hypercholesterolemia, myocardial infarction, angina pectoris, heart failure, stroke, asthma, chronic obstructive pulmonary disease, arthritis or rheumatism, osteoporosis, chronic back problems, depression, cancer, chronic renal failure, Parkinson’s disease, and Alzheimer’s disease.

Among the health conditions, the presence of two or more chronic diseases was positively associated with initial insomnia (OR = 1.21; 95%CI: 1.01; 1.45), intermediate insomnia (OR = 1.65; 95%CI: 1.32; 2.05), late insomnia (OR = 1.37; 95%CI: 1.12; 1.69), any type of insomnia (OR = 1.49; 95%CI: 1.21; 1.84), and poor sleep quality (OR = 2.21; 95%CI: 1.44; 3.40) (Table 3). Regular self-rated health was positively associated with initial insomnia (OR = 1.69; 95%CI: 1.36; 2.09), intermediate insomnia (OR = 1.88; 95%CI: 1.50; 2.37), late insomnia (OR = 1.76; 95%CI: 1.42; 2.19), any type of insomnia (OR = 1.80; 95%CI: 1.46; 2.22), poor sleep quality (OR = 2.07; 95%CI: 1.57; 2.74), and daytime sleepiness (OR = 2.00; 95%CI: 1.58; 2.52) (Table 3). Similarly, bad/very bad self-rated health was positively associated with initial insomnia (OR = 3.00; 95%CI: 2.30; 3.91), intermediate insomnia (OR = 3.34; 95%CI: 2.55; 4.38), late insomnia (OR = 3.45; 95%CI: 2.66; 4.47), any type of insomnia (OR = 3.25; 95%CI: 2.49; 4.25), poor sleep quality (OR = 4.97; 95%CI: 3.39; 7.29), and daytime sleepiness (OR = 3.18; 95%CI: 2.43; 4.16) (Table 3).

**Figure 2:**
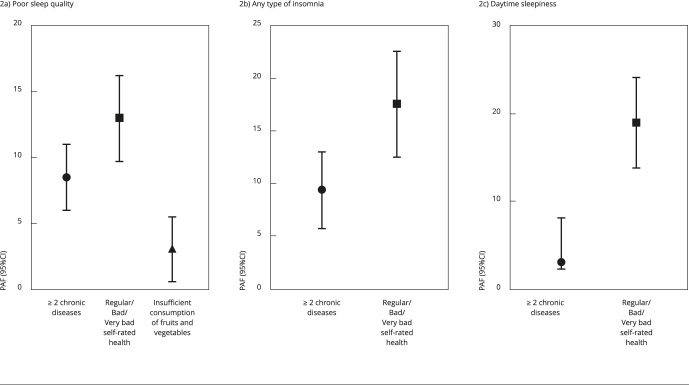
Population-attributable fraction (PAF) for potentially modifiable associated factors of sleep problems in older adults (≥ 60 years). *Brazilian Longitudinal Study of Aging* (ELSI-Brazil), 2019-2021.

For any type of insomnia, PAF was 9.4% (95%CI: 5.7; 13.0) for two or more chronic diseases and 17.6% (95%CI: 12.5; 22.6) for regular/bad/very bad self-rated health. Regarding poor sleep quality, PAF was 3.1% (95%CI: 0.6; 5.5) for insufficient consumption of fruits and vegetables, 8.5% (95%CI: 6.0; 11.0) for two or more chronic diseases, and 13% (95CI%: 9.7; 16.2) for regular/bad/very bad self-rated health (Figure 2). The PAF estimates for daytime sleepiness were 3% (95%CI: 2.2; 8.1) for two or more chronic diseases and 19% (95%CI: 13.8; 24.2) for regular/bad/very bad self-rated health (Figure 2).

95%CI: 95% confidence interval.

## Discussion

This study shows that the most prevalent sleep problems in older Brazilian adults were different typologies of insomnia (45.9% to 58.6%) followed by daytime sleepiness (38.4%) and poor sleep quality (15.6%). Moreover, we observed that being female, having two or more self-reported chronic diseases, not consuming the recommended amount of fruits and vegetables, and having regular and bad/very bad self-rated health were positively associated with almost all typologies of sleep problems investigated. Conversely, the consumption of alcohol once a month or more presented an inverse association with initial insomnia.

The prevalence proportions of sleep problems observed in this study (15.6% to 58.6%) resemble those found in older adults from the Great Britain (50.3%) ^33^, China (21%) ^34^, Brazil (14.9% to 36.1%) ^3^ ^,^ ^22^, and Japan (25%) ^35^. We highlight that previous studies conducted in Brazil have not stratified the prevalence for each sleep problem ^3^ ^,^ ^22^, making it necessary to describe an updated prevalence for each type of sleep problem in the older Brazilian population.

Studies conducted with older adults in the United States, Singapore, Brazil (city of Bambuí), and China observed a prevalence of insomnia ranging from 10.3% to 55.6% (initial insomnia), 13.1% to 40.7% (intermediate insomnia), and 10.7% to 37.2% (late insomnia) ^23^ ^,^ ^36^ ^,^ ^37^ ^,^ ^38^, prevalence proportions that are similar to those found in our study. Regarding poor sleep quality, a study with older Chinese adults found a prevalence (15.9%) similar to ours (15.6%) ^34^. On the contrary, the prevalence of daytime sleepiness identified in our study (38.4%) was higher compared to the proportion reported for older Japanese adults (25%) ^35^. It has been hypothesized that epigenetic difference plays a role in the genes that regulate the circadian cycle in Brazilian and Japanese individuals ^39^. The PER3 and CLOCK genes from Japanese individuals have a higher frequency of short alleles than genes from Brazilians, which is associated with better regulation of the circadian rhythm and, consequently, a lower occurrence of daytime sleepiness ^39^.

An upward trend in the prevalence of sleep problems has been observed in recent years ^40^. Data from the National Sleep Foundation (United States) suggest that older adults get 7-8 hours of sleep ^41^. However, about 35% of older American adults sleep less than the recommended seven hours, which can result in daytime sleepiness and other sleep problems ^42^. Furthermore, the increased prevalence of sleep problems in older adults in the last decade may be associated with the rising use of smartphones before bedtime, which increases sleep latency and leads to initial insomnia ^43^.

In our study, among the sociodemographic characteristics, only the female sex was associated with sleep problems. Previous studies have shown that the female sex increases the odds of any type of insomnia (OR = 1.58; 95%CI: 1.35; 1.85) ^44^, poor sleep quality (OR = 1.88; 95%CI: 1.54; 2.28) ^45^, and daytime sleepiness (OR = 1.40; 95%CI: 1.00; 2.06) ^46^. Evidence indicates that women have a different sleep architecture compared to men, characterized by slower wave sleep, more night awakenings, and longer sleep latency ^47^. Hormonal variation, observed especially in women, may deregulate a circuit called the orexin/hypocretin system, which is modulated by gonadal hormones and plays a key role in regulating the sleep-wake cycle ^48^. Moreover, blood cortisol level tends to be higher in women than in men ^49^, which can alter the secretion of corticotrophin-releasing factor, adrenocorticotrophic hormone, and norepinephrine and disrupt the sleep-wake cycle, leading to poor sleep quality ^50^. Another possible explanation is that most household chores are done by women, who sleep on average only six hours per night, which is considered insufficient for the maintenance of life ^51^ and contributes to daytime sleepiness due to fatigue ^46^.

Among the behavioral variables, failure to adequately consume fruits and vegetables was positively associated with poor sleep quality. Several studies have reported that fruit and vegetable intake is critical for improving sleep quality ^52^ ^,^ ^53^ ^,^ ^54^ ^,^ ^55^. In older adults from South Africa, not eating fruits and vegetables increased the odds of poor sleep quality by 1.76 (95%CI: 1.00; 3.08) ^55^, corroborating the findings of our study (OR = 1.29; 95%CI: 1.03; 1.62). This relationship may occur since fruits/vegatables are important sources of antioxidants, polyphenols, carotenoids, vitamin C, fiber, potassium, flavonoids, and other biologically active compounds, which have been proven to act through numerous pathways to control body homeostasis and play an important role in regulating circadian rhythm, thus improving sleep quality ^56^. Furthermore, fruit/vegetable intake plays an important role in modulating the metabolism and concentration of steroid hormones, which are largely related to sleep quality ^57^.

In this study, alcohol consumption once a month or more was found to be negatively associated with initial insomnia (OR = 0.72; 95%CI: 0.53; 0.97), which is consistent with the findings of Britton et al. ^58^. These authors observed that the consumption of 1-21 units (8g of alcohol per unit) was associated with lower odds for initial insomnia (OR = 0.39; 95%CI: 0.19; 0.81). It is thought that drinking low concentrations of alcohol per month may be helpful for insomnia since it induces feelings of relaxation and sleepiness, thus reducing sleep latency ^59^. However, alcohol tends to reduce the rapid eye movement (REM) sleep phase and misalign the sleep-wake cycle. These effects can result in poor sleep quality and daytime sleepiness, impacting overall well-being. Thus, it is important to consider the impact of alcohol consumption on sleep when addressing sleep-related issues and promoting healthy sleep habits ^59^. Furthermore, some alcoholic beverages, such as red wine, are rich in flavonoids, such as resveratrol, which have antioxidant properties and neuroprotective effects, improving sleep quality ^60^ ^,^ ^61^.

Regarding health conditions, we highlight that, in our study, the presence of two or more chronic diseases increased by 1.21 to 1.65 times the odds of older adults presenting initial, intermediate, late insomnia, and any type of insomnia, which is in line with previous studies that showed that the occurrence of two simultaneous chronic diseases is positively associated with initial insomnia, intermediate, and late insomnia in older adults from Germany and China ^37^ ^,^ ^62^. The concomitant presence of two or more chronic diseases has also been positively associated with poor sleep quality in older Chinese and Canadian adults ^63^ ^,^ ^64^. It is known that chronic non-communicable diseases elevate basal levels of C-reactive protein, interleukin 6, and fibrinogen, as well as biomarkers involved in regulating the inflammatory cascade related to insomnia ^65^. Moreover, medications for the treatment of chronic diseases, such as bronchodilators, beta-blockers, central nervous system stimulants, and cardiovascular agents can lead to insomnia due to the dysregulation of inflammatory cascades and their respective mechanisms of action ^37^ ^,^ ^66^.

Our results also revealed that regular and bad/very bad self-rated health were associated factors for all typologies of sleep problems in older Brazilian adults. Previous studies have shown that regular/bad/very bad self-rated health increases the odds of poor sleep quality in Brazilian adults (OR = 1.61; 95%CI: 1.32; 1.97) ^67^ and daytime sleepiness in older Brazilian adults (OR = 1.54; 95%CI: 1.06; 2.24) ^68^. However, to our knowledge, no studies have assessed the association between regular and bad/very bad self-rated health and the different typologies of insomnia. We highlight that negative self-rated health is associated with stress and anxiety, which contribute to changes in sleep quality, such as increased sleep latency, which can lead to initial insomnia ^69^ ^,^ ^70^. Negative self-rated health is often accompanied by a diagnosis of depression, which is widely known to lead to shortened REM, reduced sleep latency, reduced non-REM sleep, and increased frequency of nighttime awakenings, contributing to the development of intermediate insomnia and late insomnia, as well as daytime sleepiness ^71^ ^,^ ^72^. It should also be highlighted that changes in sleep architecture due to the aging process, such as shorter duration of deep sleep, may contribute to greater sleep fragmentation, more complaints of insomnia, greater daytime sleepiness, and consequently worse self-rated health. In this context, understanding the age-related alterations in sleep architecture is crucial for addressing issues with older adults and developing targeted interventions to improve sleep quality and overall well-being ^73^.

The strengths of this study include its large sample size and data from a nationally representative study, as well as information on the prevalence and associated factors for different sleep problems among older adults. Furthermore, this study is pioneer in Brazil by investigating several potential factors associated with different type of sleep problems using a hierarchical analytical model. In addition, to the best of our knowledge, this is the first study to investigate the PAF analysis of different sleep problems, which is a useful measure for public health since it estimates the proportion of disease or health outcome occurrence in a population that can be attributed to a specific risk factor or exposure variable.

In the present study, regular/bad/very bad self-rated health showed the highest PAF in the context of the investigated sleep problems, indicating that this exposure variable presents a substantial impact on the occurrence of sleep problems in this population. This result highlights the importance of considering individuals’ subjective perception of health when assessing and addressing sleep-related issues. To mitigate the effects of negative self-rated health on sleep problems, it is crucial to implement strategies focusing on modifiable risk factors that improve self-rated health in primary care settings ^74^. One approach could involve the establishment of physical activity groups to promote regular exercise and social interaction among participants ^75^ since physical activity has been associated with improved overall health and sleep quality. Additionally, promoting healthy nutrition habits and providing education on the importance of a balanced diet can contribute to better self-rated health and, consequently, reduce the occurrence of sleep problems ^75^.

Despite these strengths, our results should be interpreted with caution due to some limitations, among which is the cross-sectional nature of the study, which is subject to reverse causality in the association between the independent variables and outcomes. Furthermore, although a recent study showed interesting data about the behavior of ethnicity and skin color on the prevalence of sleep problems ^21^, we did not investigate the relationship between sleep problems and this sociodemographic variable. One should also consider that the variables were obtained by self-report which is subject to memory and social desirability bias. In addition, our outcomes were not collected using standardized scales. However, self-report has been commonly used to assess the presence of sleep problems in recent studies ^76^ ^,^ ^77^. Finally, the occurrence of naps during the day was not investigated in this study since it was not evaluated in the ELSI-Brazil survey. This variable is extremely relevant and should be investigated in future studies as its occurrence is a frequent practice among older adults and its duration and time can influence sleep latency at night and nocturnal awakenings. Further studies should be conducted in this population to examine the longitudinal associations of sociodemographic and behavioral characteristics and health conditions with sleep problems to provide a higher level of evidence for therapeutic approaches and inputs for public health policy.

## Conclusions

The findings of this study reveal a high prevalence of sleep problems among older Brazilian adults, emphasizing the need for targeted public health interventions to address this issue. Additionally, we found that being female, having two or more chronic diseases, not consuming the recommended amount of fruits and vegetables, and having regular and bad/very bad self-rated health were associated with higher odds of presenting the investigated sleep problems. Furthermore, regular and bad/very bad self-rated health showed the highest PAF in the context of the investigated sleep problems.

These results also provide valuable insights for informing public health policies and strategies aimed at promoting better sleep health in this population. One key implication of this study is the importance of implementing informational campaigns and educational initiatives to raise awareness about sleep problems among older adults. Public health services should develop targeted interventions that provide information on sleep hygiene practices, the impact of sleep problems on overall health, and the available resources for seeking assistance. By disseminating accurate and accessible information, individuals can be informed to recognize, manage, and seek appropriate treatment for their sleep problems.
